# The Effect of Aging Treatment on the Properties of Cold-Rolled Cu-Ni-Si-Co Alloys with Different Mg Contents

**DOI:** 10.3390/ma18143263

**Published:** 2025-07-10

**Authors:** Dan Wu, Jinming Hu, Qiang Hu, Lingkang Wu, Bo Guan, Siqi Zeng, Zhen Xing, Jiahao Wang, Jing Xu, Guojie Huang, Jin Liu

**Affiliations:** 1 Jiangxi Key Laboratory of Advanced Copper-Based Materials, Institute of Applied Physics, Jiangxi Academy of Sciences, Nanchang 330096, China; bird5810761@163.com (D.W.); hujinming0702@163.com (J.H.); wumengling50@126.com (L.W.); kikiclover_999@163.com (S.Z.); zhenxing201111@163.com (Z.X.); wjhyjyo@163.com (J.W.); jingxu1016@163.com (J.X.); hguojie@126.com (G.H.); ljustb@163.com (J.L.); 2 State Key Laboratory of Nonferrous Metals and Processes, GRIMAT Engineering Institute Co., Ltd., Beijing 101407, China

**Keywords:** Cu-Ni-Si-Co alloy, Mg content, precipitate, aging treatment, tensile strength, electrical conductivity

## Abstract

Cu-Ni-Si is a prominent example of a high-end lead frame copper alloy. The enhancement of strength without compromising electrical conductivity has emerged as a prominent research focus. The evolution of the precipitates exerts a significant influence on the strength and electrical conductivity of Cu-Ni-Si-Co-Mg alloys. In this paper, the effects of aging treatment and Mg addition on the properties and precipitates of cold-rolled Cu-Ni-Si-Co alloys were studied. The precipitate was (Ni, Co)_2_Si and was in a strip shape. During aging, precipitation and coarsening of the (Ni, Co)_2_Si precipitates were observed. In the early stage of aging, a significant number of fine (Ni, Co)_2_Si precipitates were formed. These fine precipitates could not only have a better effect of precipitation strengthening, but also impeded the dislocation movement, thus increasing the dislocation density and improving the dislocation strengthening effect. However, the coarsening of the precipitates became dominant with increasing aging times. Therefore, the strengthening effect was weakened. The addition of 0.12% Mg promoted finer and more diffuse precipitates, which not only improving the tensile strength by 100–200 MPa, but also exhibiting a smaller effect on the electrical conductivity. However, further increases in Mg contents resulted in a significant decrease in electrical conductivity, with little change in the tensile strength. The optimum amount of added Mg was 0.12%, and the aging parameters were 300 °C and 20 min.

## 1. Introduction

The rapid development of computer and 5G communication technologies has led to significant advancements in chip integration and functionality, resulting in increased performance demands for related equipment. IC lead frame materials, as the key materials in chip manufacturing, must now have higher strengths, electrical and thermal conductivities, elasticity and dimensional stability [1,2,3]. Copper is the ideal material for connectors and other electrical or electronic products due to its high electrical and thermal conductivity, plasticity, corrosion resistance and strength [4,5,6]. Copper alloy strips are the most used structural, conductive and heat-dissipating material in chip assembly [7]. Through continuous development, copper alloy lead frame materials have formed a series of products such as Cu-Ni-Si [8], Cu-Fe-P [9], Cu-Ni-Sn [10] and Cu-Cr-Zr [11]. Cu-Ni-Si alloy strips are of particular interest due to their high strength, medium conductivity and heat-resistant characteristics.

Since M.G. Corson first discovered the age-strengthening properties of Cu-Ni-Si alloys in 1927, the use of Cu-Ni-Si alloys as lead frame materials has developed rapidly. Research into the aging behavior and precipitate structure of this alloy has also been intensified. Product development of Cu-Ni-Si alloys is aimed at achieving alloys that combine high strength with sufficient electrical conductivity. For Cu-Ni-Si alloys, which are precipitation-strengthened alloys, the strength is greatly influenced by the number, type, size and homogeneity of the precipitates [12,13]. These precipitates have been observed to interact with dislocations and solute atoms in the matrix, thereby impeding the dislocation motion and increasing the alloy strength. The electrical conductivity of Cu-Ni-Si alloys is principally influenced by the composition and type of the solute atoms that are present within the matrix [14,15]. Distortions in the copper matrix lattice may be caused by the presence of solute atoms with differing atomic radii relative to those of the copper atoms. The magnitude of the discrepancy between the atomic radii of the solute atoms and the copper atoms has been demonstrated to correlate directly with the extent of lattice distortion. In turn, this results in an increase in the scattering effect on electron movement, thereby reducing the electrical conductivity of the alloy.

The full precipitation of Ni and Si in Cu-Ni-Si alloys is pivotal to balancing high strength and high electrical conductivity. Previous studies have principally been conducted by means of adjusting the Ni/Si ratio in the alloy [16,17] in combination with suitable aging formulations [18,19]. Li et al. [16] investigated nine Cu-Ni-Si alloys with different Ni/Si ratios. The results demonstrated that the alloys exhibited increased hardness when the Ni/Si ratio ranged from 3.6 to 5.1 and enhanced electrical conductivity when the Ni/Si ratio ranged from 4.2 to 6.2. It was concluded that the Ni/Si ratio was optimized at a value of 4–5. In this instance, the full precipitation of Si and Ni atoms is possible, thus resulting in the optimal strengthening effect and enhanced electrical conductivity. Ahn, J.H., et al. [18] used prolonged aging to form layered discontinuous precipitates (DPs). A wide range of strength and electrical conductivity values can be obtained by varying the aging time, thereby enabling control of the ratio of continuous to discontinuous precipitates (CPs). Cao et al. [19] accelerated discontinuous precipitation by applying pre-deformation to Cu-6Ni-1.42Si alloy, thereby reducing the aging time from 24 h to 30 min. This provides a novel approach to high-performance conductive materials.

However, the performance of ternary Cu-Ni-Si alloys is gradually approaching the limit, and the space for development is diminishing. Consequently, researchers have gradually added various alloying elements into Cu-Ni-Si system, including Co [20,21], Cr [22], Al [23], Ti [24], P [25] and Mg [8,26,27]. The addition of Co leads to the formation of (Ni, Co)_2_Si precipitates. It precipitates easily and is more difficult to roughen than δ-Ni_2_Si [28]. Cr has a significant impact on the nucleation rate of second-phase particles in Cu-Ni-Si alloys. However, the average particle size is also found to increase [22]. The addition of Al or P has been demonstrated to refine the microstructure of Cu-Ni-Si alloys, promote the precipitation of Ni_2_Si phase, and effectively improve the strength and hardness of the alloy [23,25]. The addition of Ti in Cu-Ni-Si alloys has been shown to reduce the grain size and increase the elongation considerably but accelerate the coarsening of the Ni_2_Si phase [24]. Mg has been shown to play a significant role in the reduction in precipitation spacing of the precipitates. In addition, Mg atoms have been observed to exert a drag effect on dislocation motion, thereby enhancing the strength and resistance to stress relaxation [8]. Considering that the (Ni, Co)_2_Si phase is comparatively more difficult to roughen than the δ-Ni_2_Si phase, this study focuses on a Cu-Ni-Si-Co alloy as the primary research object. In addition, the addition of Mg is intended to induce the precipitation of the (Ni, Co)_2_Si phase, with a view to enhancing the strength and electrical conductivity of Cu alloys. However, there are few studies related to the Cu-Ni-Si-Co-Mg alloy system, particularly with regard to the evolutionary behavior of the precipitates in Cu-Ni-Si-Co alloys with the addition of Mg. It is therefore necessary to explore the effect of Mg addition on the precipitates and properties in the Cu-Ni-Si-Co alloy system in order to provide guidance for the subsequent development of new alloy systems.

The present study is based on Cu-Ni-Si-Co alloys with different Mg contents. The effects of aging time, temperature and Mg content on the micromorphology and evolutionary behavior of the precipitates were analyzed. The dislocation densities of various alloys were calculated theoretically. The relationship between the precipitates and the properties was established, and the appropriate parameters for the heat treatment process were determined.

## 2. Materials and Methods

**Figure 1:**
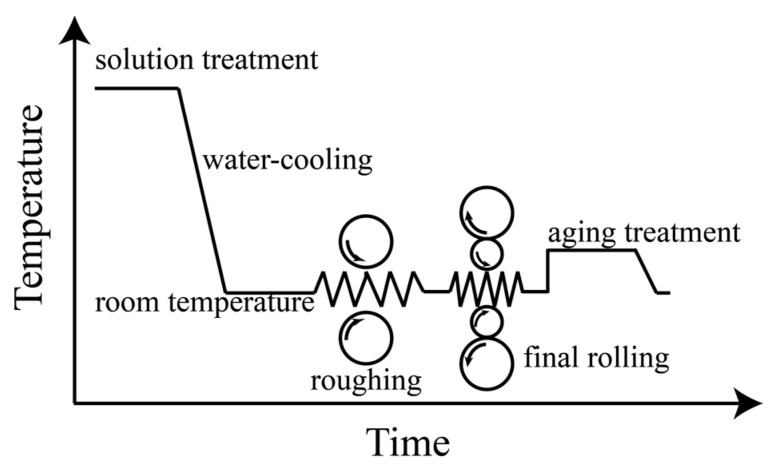
Schematic diagram of rolling and heat treatment process.

Cast Cu-1.86Ni-1.1Co-0.6Si-xMg alloys (x = 0, 0.12%, 0.24%) with dimensions of 60 mm × 30 mm × 20 mm were prepared by vacuum induction furnace under an argon atmosphere. The raw materials for smelting were pure copper (99.99%), pure nickel (99.99%), pure silicon (99.99%), pure cobalt (99.99%) and pure magnesium (99.99%). The elemental compositions in different copper alloy ingots were examined by an ICP spectrometer (Plasma 2000, NCS Testing Technology Co., Ltd., Beijing, China), as shown in Table 1. The schematic diagram of the rolling and heat treatment process is shown in Figure 1. Before rolling, the samples were subjected to solution treatment, held at 850 °C for 5 h, and then water-quenched. Then, the samples were first rolled on a two-roll mill (diameter 350 mm × 400 mm) at 90% total deformation. The ingot was first rolled from 20 mm to 10 mm, and then from 10 mm to 2 mm, with a reduction of 20% per pass. The samples were finally rolled on the experimental four-roll mill (diameter 90 mm × 420 mm/350 mm × 400 mm). The ingots were rolled from 2 mm to 0.45 mm and finally to 0.2 mm, with a reduction of 10% per pass. After rolling, the thin plate samples with dimensions of 100 mm × 10 mm × 0.2 mm were subjected to an aging treatment in an air atmosphere at three different temperatures: 300 °C, 350 °C and 400 °C. The aging times for each group of experiments were 10, 20, 30 and 40 min, respectively.

**Figure 2:**
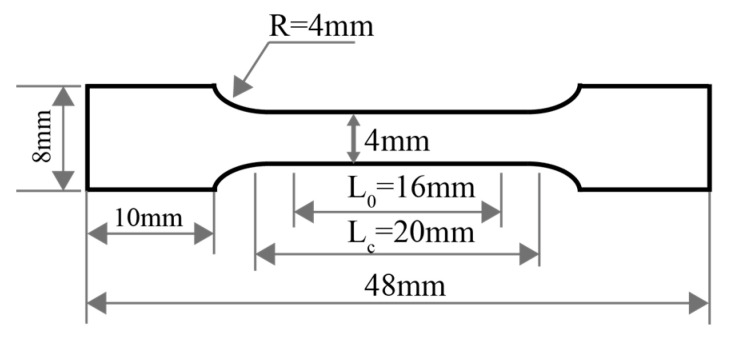
Schematic diagram of tensile sample.

The microstructure and precipitates of the samples were observed by high-resolution field emission scanning electron microscopy with a secondary electrons detector (SE) and EDS (FE-SEM, GeminiSEM 460, ZEISS, Oberkochen, Germany). The SEM samples were first polished with sandpaper until the surface was smooth and then electrolytically polished using a solution of nitric acid and methanol at a ratio of 2:8, with a voltage of 20 V, a current of approximately 0.8 A and a polishing time of around 20 s. The size, morphology, distribution and chemical composition of the precipitates were also investigated. The number of precipitates was calculated for each sample using three independent fields of view, each measuring 22.6 μm × 15.6 μm. ImageJ software (1.49v) was used to measure the size of the precipitates, and the average size was obtained by calculating the arithmetic mean. The morphologies and diffraction patterns of the precipitates were studied by transmission electron microscopy (TEM, JEM-F200, JEOL, Tokyo, Japan). The TEM samples were first sanded to a thickness of approximately 100 μm and then cut into 3 mm diameter disks. The thin disks were then ion-polished using an ion polishing system (Gatan 695, Gatan Inc., Pleasanton, CA, USA) at an accelerating voltage of 1–6 kV and at a temperature of −70 °C. The phase composition of the samples was analyzed using an XRD diffractometer (Ruker D8 Advance, Bruker AXS GmbH, Karlsruhe, Germany) with a scan angle of 40–100° (2θ) and a scan speed of 2°/min. The electrical conductivity of the samples with dimensions of 100 mm × 10 mm × 0.2 mm (±0.01) was measured and converted by means of a digital micro-ohmmeter (ZY9987, Shanghai Zhengyang Instrument Factory, Shanghai, China). The dimensions of the tensile specimen complied with the Chinese standard GB/T 228.1-2021 [29], as illustrated in Figure 2. The strips with a thickness of 0.2 mm (±0.01) were processed by wire electrical discharge machining, and 3 tensile specimens of each sample were tested. The tensile strength of the samples was tested using an electronic universal testing machine (Meters CMT5205, MTS Systems (China) Co., Ltd., Shanghai, China) with a tensile rate of 2 mm/min at room temperature.

## 3. Results

### 3.1. Conductivity and Strength

**Figure 3:**
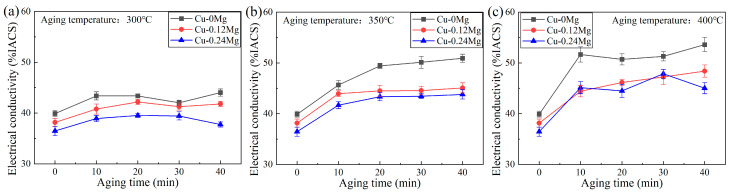
The electrical conductivities of different samples: (**a**) 300 °C; (**b**) 350 °C; (**c**) 400 °C.

Figure 3 shows the electrical conductivities of different samples. There is a gradual increase in the electrical conductivity as the aging time increases. The higher the aging temperature is, the greater the increase in conductivity is. At an aging temperature of 300 °C, the electrical conductivity increases less with increasing aging times. The addition of Mg reduces the electrical conductivity. For example, the electrical conductivities of the Cu-0Mg, Cu-0.12Mg and Cu-0.24Mg samples after 20 min of aging are 43.4% IACS, 42.2% IACS and 39.5% IACS, respectively. Compared with the Cu-0Mg sample, the addition of 0.12%Mg reduces the electrical conductivity by 1.1% IACS, whereas adding 0.24%Mg decreases it by 3.9%. That is, adding 0.24% Mg causes a rapid decrease in conductivity. At an aging temperature of 350–400 °C, the electrical conductivity increases rapidly with increasing aging times. For the Cu-0.12Mg sample, the conductivity increases by 3.6%, 6.9%, and 10.2% IACS, respectively, after being aged for 40 min at 300 °C, 350 °C and 400 °C. The electrical conductivity reaches 45% IACS at an aging temperature of 400 °C.

**Figure 4:**
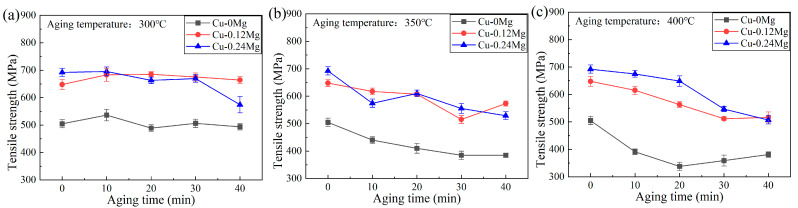
The tensile strengths of different samples: (**a**) 300 °C; (**b**) 350 °C; (**c**) 400 °C.

The tensile strengths of different samples are shown in Figure 4. For the Cu-0.12Mg-300 °C samples, the tensile strength appeared to increase and then decrease with increasing aging times, increasing from 648 MPa (0 min) to 685 MPa (20 min) and then decreasing to 664 MPa (40 min).

A comparison of copper alloys with different Mg contents shows that the addition of Mg has a major influence on the tensile strength. The tensile strength is increased by more than 150 MPa after the addition of 0.12%Mg. In particular, the tensile strength of the alloy increases from 488 MPa to 685 MPa after being aged at 300 °C for 20 min. However, the tensile strength of the Cu-0.24Mg sample does not differ significantly from that of the Cu-0.12Mg sample. The tensile strengths of the Cu-0.12Mg and Cu-0.24Mg samples after being aged at 300 °C for 20 min are 685 MPa and 663 MPa. Coupled with previous electrical conductivity studies, it is shown that adding 0.24% Mg makes it difficult to further increase the tensile strength, while reducing the electrical conductivity. At an aging temperature of 350–400 °C, the tensile strength of the samples decreases gradually as the aging time increases. For the Cu-0.12Mg sample, the tensile strength decreases by 75 MPa and 132 MPa, respectively, after being aged for 40 min at 350 °C and 400 °C.

**Figure 5:**
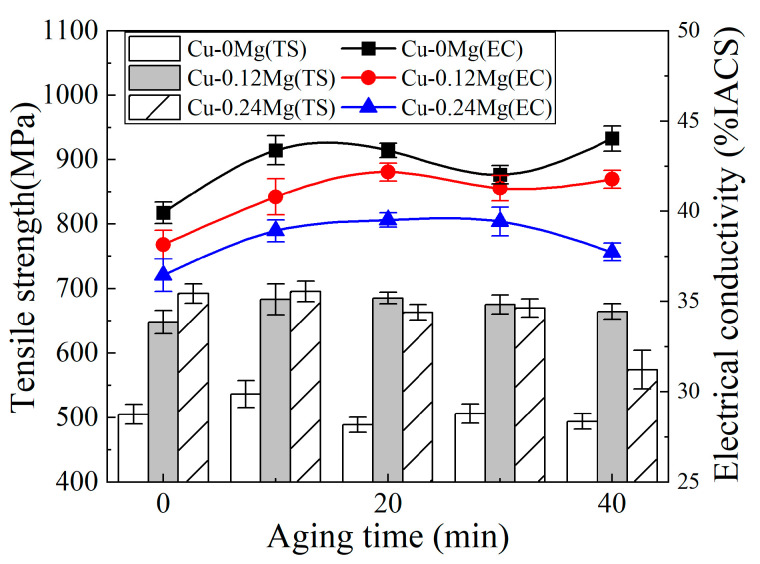
The electrical conductivities and tensile strengths of different samples after aging at 300 °C.

The electrical conductivities and tensile strengths of different samples after aging at 300 °C are shown in Figure 5. The Cu-0.12Mg-300 °C-20 min sample has the best performance in terms of overall electrical conductivity and tensile strength. This means that the optimum amount of added Mg is 0.12%, and the aging parameters are 300 °C and 20 min.

### 3.2. Precipitate Observation

**Figure 6:**
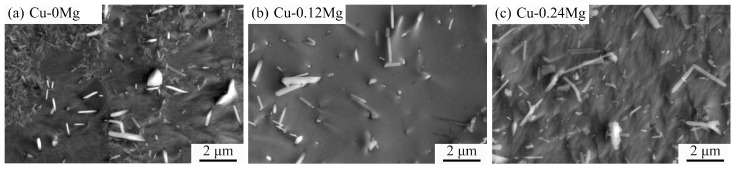
The micromorphologies and precipitates of different samples after solution treatment: (**a**) Cu-0Mg sample; (**b**) Cu-0.12Mg sample; (**c**) Cu-0.24Mg sample.

The micromorphologies and precipitates of different samples after solution treatment are shown in Figure 6. There are some large precipitates in the copper alloy. These are primary precipitates that are formed during solidification and solution treatment, which are approximately 1–3 um in length [30]. For the Cu-0Mg and Cu-0.12Mg samples, most of the smaller precipitates have been dissolved into the copper matrix, leaving only a small amount behind. However, the Cu-0.24Mg sample contains a large number of precipitates and exhibits relatively severe segregation, which may affect precipitation during aging.

**Figure 7:**
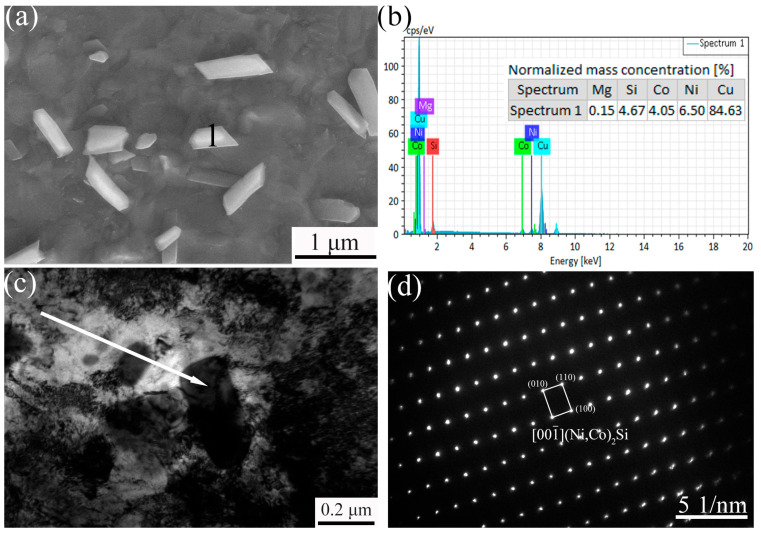
The micromorphology and analysis of precipitates in Cu-0.12Mg sample aged at 300 °C for 40 min: (**a**) SEM; (**b**) EDS; (**c**) TEM; (**d**) diffraction pattern.

The micromorphology and compositional analysis of the precipitates in the Cu-0.12Mg sample after aging treatment are shown in Figure 7. As demonstrated in Figure 7a,b, the SEM images and EDS analysis of the samples reveal that the precipitates manifest as strips with sizes ranging from 50 to 500 nm, which are mainly precipitated during the rolling and aging processes [31]. The EDS results indicate that the main compositions of the precipitates are Ni, Co, Si and Cu. Figure 7c,d show the TEM images and diffraction patterns of the precipitates, respectively. The diffraction patterns obtained are similar to those of the δ-Ni_2_Si (orthorhombic structure, a = 0.703 nm, b = 0.499 nm, c = 0.372 nm) [32]. It has been demonstrated that the precipitates are δ-(Ni, Co)_2_Si [33], as a result of the substitution of some Ni atoms in δ-Ni_2_Si with Co.

**Figure 8:**
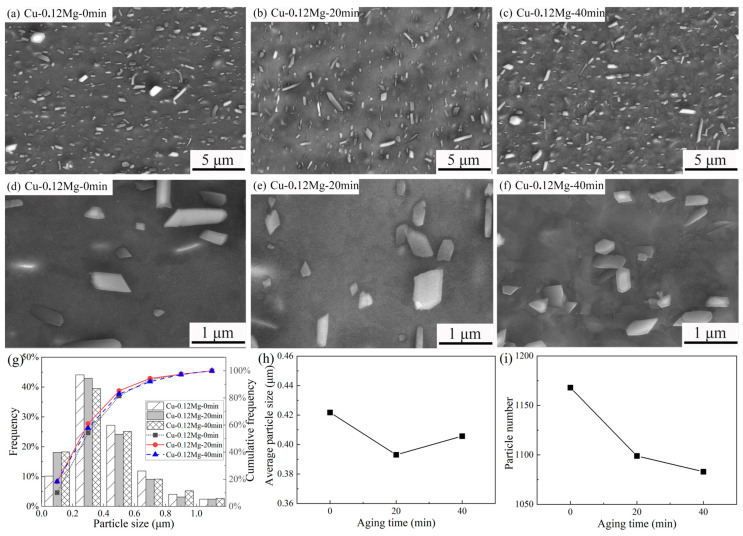
The micromorphologies and statistical analyses of the precipitates in Cu-0.12Mg samples after aging at 300 °C for different durations: (**a**,**d**) Cu-0.12Mg-0 min sample; (**b**,**e**) Cu-0.12Mg-20 min sample; (**c**,**f**) Cu-0.12Mg-40 min sample; (**g**) particle size frequency distributions; (**h**) average particle size; (**i**) particle number.

Figure 8 shows the micromorphologies and statistical analyses of the precipitates in Cu-0.12Mg samples after being aged for different durations. Comparing Figure 8a–c with Figure 6b, a large number of small precipitates emerge after cold rolling and aging. The Cu-0.12Mg-20 min sample contains a greater number of small precipitates than the Cu-0.12Mg-0 min sample, as shown in Figure 8d,e. This indicates that small precipitates are gradually formed during the aging process. By contrast, the number of small precipitates in the Cu-0.12Mg-40 min sample is similar to that in the Cu-0.12Mg-20 min sample.

The size and number of precipitates were calculated by selecting the same number and visual fields as shown in Figure 8g–i. For the Cu-0.12Mg samples, the proportions of precipitates smaller than 200 nm after aging for 0, 20 min and 40 min are 10.2%, 18.1% and 18.3%, respectively. This indicates that a large number of small (Ni, Co)_2_Si were precipitated during aging, which enhances the effect of precipitation strengthening. However, the proportions of precipitates smaller than 400 nm drops from 61.1% to 57.8% as the aging time increases from 20 min to 40 min. Therefore, Figure 8h shows that the average particle size of the Cu-0.12Mg samples after aging for 0, 20 min and 40 min are 0.42 μm, 0.39 μm and 0.41 μm, respectively, i.e., the average particle size is smallest following aging for 20 min. The particle numbers of the Cu-0.12Mg samples after aging for 0, 20 min and 40 min are 1168, 1099 and 1083, respectively, as shown in Figure 8i. In combination with Figure 8g–i, it is found that small (Ni, Co)_2_Si is gradually precipitated, and the already precipitated (Ni, Co)_2_Si is also roughened. These two phenomena occur simultaneously. In the early stage of aging, a large number of small (Ni, Co)_2_Si precipitates are formed, and the proportion of small precipitates increases. Although accompanied by a coarsening of (Ni, Co)_2_Si, the average particle size decreases. However, the coarsening of the precipitates begins to dominate as the aging time increases. The proportion of small precipitates does not change much, so the average particle size increases, and the precipitation strengthening reduces.

**Figure 9:**
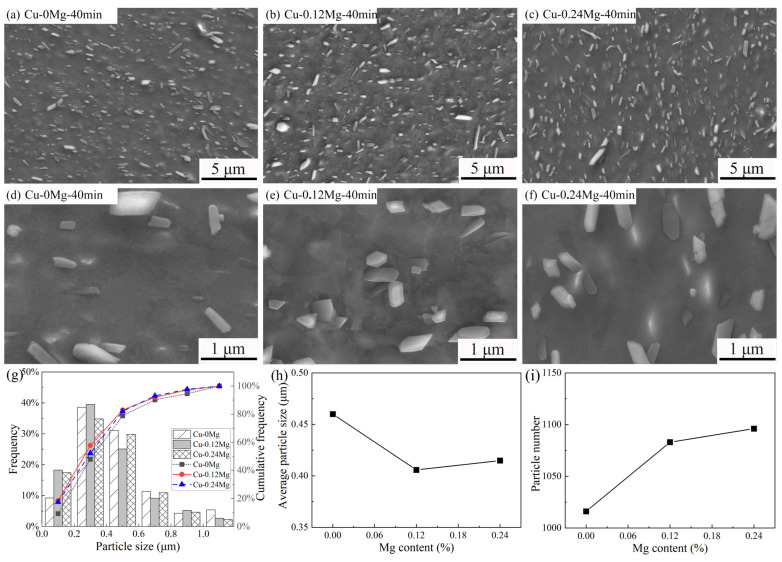
The micromorphologies and statistical analyses of the precipitates in different samples after aging at 300 °C for 40 min: (**a**,**d**) Cu-0Mg sample; (**b**,**e**) Cu-0.12Mg sample; (**c**,**f**) Cu-0.24Mg sample; (**g**) particle size frequency distributions; (**h**) average particle size; (**i**) particle number.

The micromorphologies and statistical analyses of different samples are shown in Figure 9. The same large primary precipitates, approximately 1–3 um long, are present in the matrix of the Cu-0Mg samples. Compared to the Cu-0.12Mg sample, the number of precipitates, especially small precipitates, in the Cu-0Mg sample is relatively low, as shown in Figure 9d,e. This suggests that the addition of 0.12%Mg mainly promotes the precipitation of small (Ni, Co)_2_Si, which has a better strengthening effect compared to large precipitates. However, a slight increase in precipitate content is observed as the Mg content is further increased, as shown in Figure 9e,f.

As can be seen from Figure 9g–i, the proportions of precipitates smaller than 200 nm of the Cu-0Mg, Cu-0.12Mg and Cu-0.24Mg samples are 9.3%, 18.3% and 17.4%, respectively. Therefore, the average particle size of Cu-0.12Mg samples is the smallest. Meanwhile, the proportions of precipitates smaller than 400 nm drops from 57.8% to 52.3% as the Mg content increases from 0.12 to 0.24%. That is, the addition of 0.24%Mg has less effect on the precipitation of small (Ni, Co)_2_Si. Figure 9i also shows that the numbers of precipitates in the Cu-0Mg, Cu-0.12Mg and Cu-0.24Mg samples are 1016, 1083 and 1096, respectively. The number of precipitates increases significantly when the Mg content increases from 0 to 0.12%. However, the rate of increase is slower with further increases in Mg content. In combination with the analysis presented in Figure 9h, the addition of Mg can reduce the precipitation activation energy of (Ni, Co)_2_Si and facilitate the precipitation of the second phase [8]. Therefore, adding 0.12%Mg promotes the precipitation of small precipitates, which increases the number of precipitates and decreases the average particle size.

**Figure 10:**
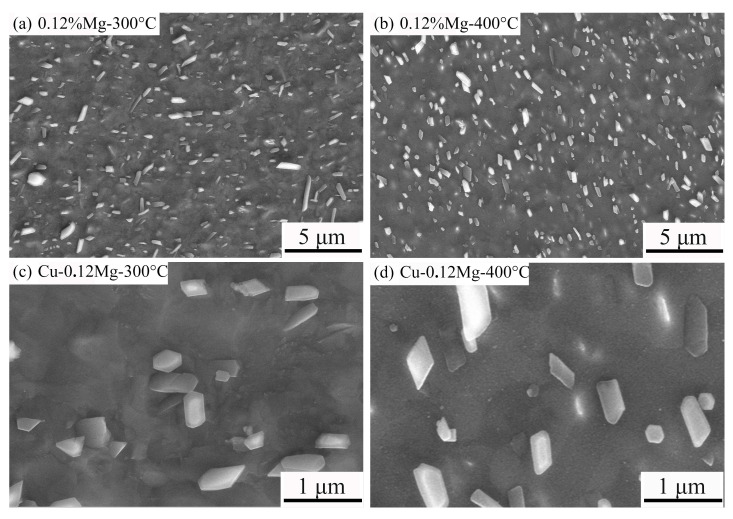
The micromorphologies of the precipitates in the Cu-0.12Mg samples after aging at different temperatures for 40 min: (**a**,**c**) Cu-0.12Mg-300 °C sample; (**b**,**d**) Cu-0.12Mg-400 °C sample.

Figure 10 shows the micromorphologies of the precipitates in the Cu-0.12Mg samples after aging at different temperatures for 40 min. The Cu-0.12Mg-400 °C sample has a higher number of precipitates than the Cu-0.12Mg-300 °C sample. And the majority of the precipitates in the Cu-0.12Mg-400 °C sample are around 400 nm in size. At higher aging temperatures, the precipitation rate of the precipitates is faster. Therefore, the electrical conductivity increases rapidly as large amounts of Ni, Co and Si elements precipitate from the matrix. But the precipitates are more prone to coarsening, and the strengthening effect is reduced. A comparison of Figure 10c,d reveals that while the total amount of precipitates increases with increasing aging temperatures, the number of small precipitates does not increase. This finding suggests that the coarsening behavior of the precipitates is more pronounced at elevated temperatures.

## 4. Discussion

### 4.1. Dislocation Analysis

**Figure 11:**
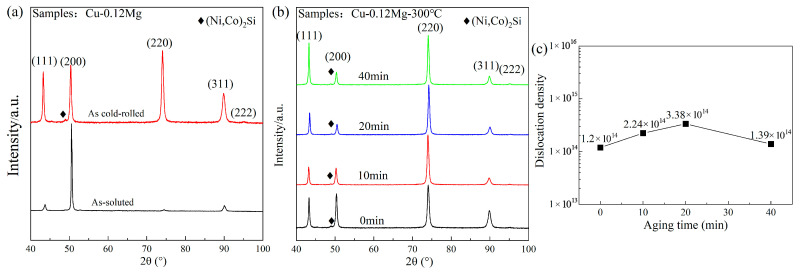
The XRD analysis and dislocation densities of the Cu-0.12Mg samples: (**a**) XRD analysis of the solid solution and cold-rolled state samples; (**b**) XRD analysis of samples aged at 300 °C for different durations; (**c**) dislocation density.

Figure 11 shows the XRD analysis and dislocation density of the Cu-0.12Mg samples. As shown in Figure 11a, the Cu (200) peak dominates in the diffractogram for solid solution samples, while others, such as (111), (220) and (311), are relatively weak. No diffraction peaks were observed for (Ni, Co)_2_Si. This indicates that the (Ni, Co)_2_Si phase mostly dissolves into the copper matrix at high temperatures, as shown in Figure 6b. After cold rolling and aging, the (111), (220) and (311) diffraction peaks are enhanced, with the (220) peak becoming the strongest and the (222) peak appearing, as shown in Figure 11b. And the diffraction peaks of (Ni, Co)_2_Si can also be seen, suggesting that a significant quantity of (Ni, Co)_2_Si precipitates form following cold rolling and aging.

In the present study, the dislocation density of copper alloys can be calculated from the modified Williamson–Hall formula and XRD measurements, [34,35,36,37] as follows:

Δ*K* = α + M · b ((π · *ρ*)/2) ^1/2^ · *K* · *C*^1/2^ + *O*(*K*^2^ · *C*) (1)

where *K* = 2sin*θ/λ*, Δ*K* = 2cos*θ·*Δ*θ/λ*, *θ* and *λ* are the diffraction angle and the X-ray wavelength (0.154056 nm), respectively. 2Δ*θ* is the measured full width at half maximum (FWHM) [34]. α = 0.9/*D* and *D* is the average grain size. By fitting Δ*K* and *K* according to Equation (1) and neglecting the high exponential term (*O*), the linear intercept obtained is α. M is the dislocation distribution parameter (M = 1.4) [38]. b is the Burgers Vector (b = 0.255) [39]. *ρ* is the dislocation density. *C* is the dislocation average contrast factor, as in the following equation:

*C=C_h00_* (1-q⋅*H*^2^) (2)

*H*^2^ = (*h*^2^ · *k*^2^ + *k*^2^ · *l*^2^ + *l*^2^ · *h*^2^)/(*h*^2^ + *k*^2^ + *l*^2^)^2^ (3)

where *C_h00_* is a constant (0.3040) [36]. q is a constant of relevance to the experiment. *h*, *k* and *l* are Miller indices. Substituting Equation (2) into Equation (1) and neglecting the high exponential term (*O*) gives the following:

(Δ*K*-*α*)^2^/*K*^2^ = (π· M^2^ · b^2^ ·*ρ*)/2 ⋅ 0.3040(1-q · *H*^2^) (4)

The dislocation density (*ρ*) can be obtained from the linear intercept of(Δ*K*-*α*)^2^/*K*^2^ and *H*^2^ in Equation (4).

Figure 11c shows the dislocation density of Cu-0.12Mg sample. The dislocation density of the solid solution samples is too low to obtain accurate results through calculation. The dislocation density increases from 1.2 × 10^14^/m^2^ to 3.38 × 10^14^/m^2^ as the aging time increases to 20 min. In the early stages of aging, the solid dissolved Ni, Si and Co elements in the copper alloy are precipitated in large quantities. Fine diffused (Ni, Co)_2_Si is formed. These fine second-phase particles impede dislocation motion, causing dislocations to become entangled near the precipitates and increasing the dislocation density. The SEM and XRD results show that the (Ni, Co)_2_Si precipitate in a solid solution state is large in size and small in quantity. Therefore, it has little effect on dislocations. Compared to the Cu-0.12Mg-300 °C-0 min sample, the Cu-0.12Mg-300 °C-20 min sample has better precipitation strengthening and dislocation strengthening effects, thus increasing the strength, as shown in Figure 4a. Although the strength increases, the conductivity does not decrease, rising from 38.2% IACS (0 min) to 42.2% IACS (20 min), as shown in Figure 3a. The electrical conductivity of Cu-Ni-Si alloys is most significantly influenced by the dissolved elements in the matrix [40]. Therefore, the conductivity is improved by the precipitation of solid dissolved Ni, Si and Co elements from the matrix, despite the fact that increased dislocation densities and precipitates can have a certain detrimental effect on conductivity. Instead, the dislocation density decreases from 3.38 × 10^14^/m^2^ to 1.39 × 10^14^/m^2^ as the aging time increases to 40 min. The coarsening of the precipitates begins to dominate at this stage. The hindering effect of the coarsening precipitate on the dislocations is weakened; therefore, the dislocation density and dislocation strengthening effect decrease. The dislocation density in the alloy exhibits a maximum after aging at 300 °C for 20 min.

**Figure 12:**
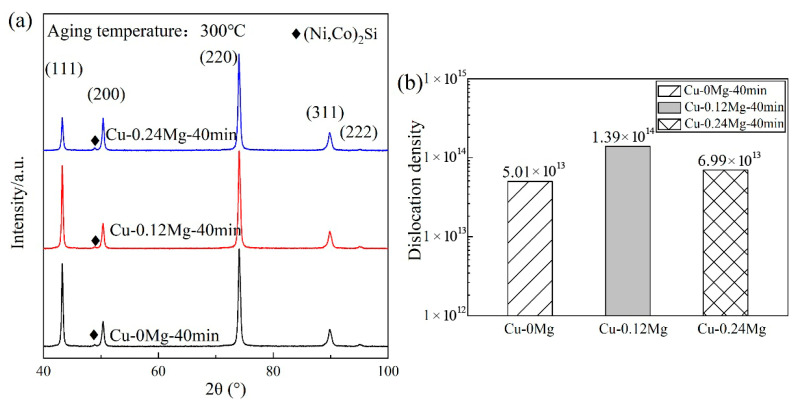
The XRD analysis and dislocation densities of different samples after aging at 300 °C for 40 min: (**a**) XRD analysis; (**b**) dislocation density.

The XRD analysis and dislocation density of different samples are shown in Figure 12. The most intense peak is observed at (220) for all copper alloys with different Mg contents. Furthermore, the diffraction peak of (Ni, Co)_2_Si is evident in all cases. As demonstrated in Figure 12b, the dislocation densities of Cu-0Mg, Cu-0.12Mg and Cu-0.24Mg samples are 5.01 × 10^13^/m^2^, 1.39 × 10^14^/m^2^ and 6.99 × 10^13^/m^2^, respectively. The Cu-0.12Mg sample had the highest dislocation density of the three samples. As previously stated, the addition of 0.12%Mg results in the precipitation of many small precipitates, which increases the hindering effect on the dislocations and increases dislocation density. Nevertheless, the addition of 0.24%Mg leads to a decrease in the proportion of small precipitates. Thus, the hindering effect on dislocations is rather diminished.

**Figure 13:**
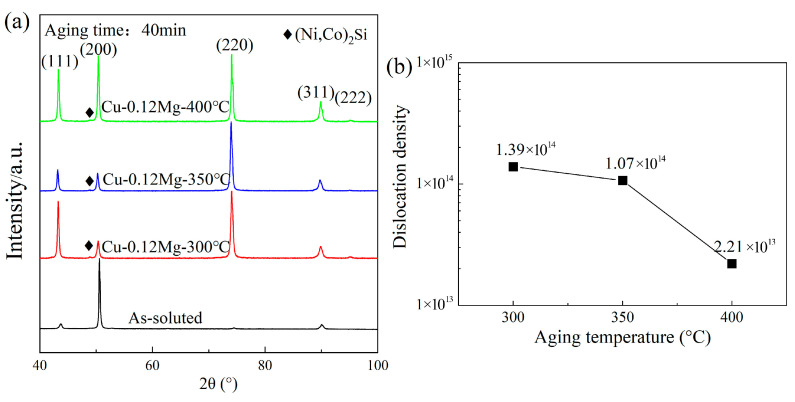
The XRD analysis and dislocation densities of Cu-0.12Mg samples: (**a**) XRD analysis of the solid solution and samples subjected to different aging temperatures; (**b**) dislocation density.

As shown in Figure 13, the strongest peak in the copper alloy shifts from the (200) peak to the (220) peak after cold rolling and low-temperature aging. After aging at 400 °C, the strongest peak in the copper alloy remains the (220) peak, although its peak value differs little from that of the (200) peak. Additionally, slight splitting of the diffraction peaks can be observed at (220) and (311), with irregular peaks appearing near the main ones. This may indicate the onset of recrystallisation phenomena. Figure 13b shows that the dislocation density decreases rapidly as the aging temperature increases, decreasing from 1.39 × 10^14^/m^2^ (300 °C) to 2.21 × 10^13^/m^2^ (400 °C), a difference of almost an order of magnitude. It shows that aging temperature has a large influence on the dislocation density. The dislocation strengthening effect is significantly reduced at higher aging temperatures. Recrystallisation of the matrix microstructure may occur at higher aging temperatures, resulting in the disappearance of the original dislocation structure.

At the same time, the coarsening phenomenon of the precipitates is evident, and the inhibition effect on the dislocation is weakened. As a result, the dislocation density in the alloy decreases rapidly. Meanwhile, the dislocation strengthening effect is greatly reduced at higher aging temperatures. As both the precipitation and dislocation strengthening effects are weakened, the strength of the copper alloy decreases rapidly, as shown in Figure 4c.

### 4.2. Effects of Mg Addition on the Precipitates and Mechanical Properties

The addition of Mg has a major influence on the precipitates and mechanical properties. As shown in Figure 4, the tensile strength is increased by more than 150 MPa after the addition of 0.12%Mg, but the electrical conductivity is reduced. Li [41] found that there was no apparent aggregation of Mg elements in Cu-Ni-Si-Cr-Mg alloys, which means that Mg is uniformly solubilized in the matrix. Therefore, the greater the addition of Mg is, the greater the damage to the electrical conductivity of the substrate is. Figure 3 also shows that Cu-0.24Mg samples have the lowest electrical conductivity. On the other hand, the addition of Mg reduces the activation energy of precipitates and increases the formation rate [8]. As shown in Figure 9, the number of precipitates increases with the addition of Mg. This suggests that more elements, such as Ni, Co and Si, are precipitated to compensate for the loss of electrical conductivity due to the solid solution of Mg in the matrix. However, the increase rate of precipitates is slow as the Mg content further increases, resulting in a faster decrease in electrical conductivity.

Studies [41,42] show that in the strengthening mechanisms of Cu-Ni-Si alloys precipitation strengthening and dislocation strengthening dominate. Figure 9 shows that the addition of 0.12%Mg increases the proportion of precipitates smaller than 200 nm from 9.3% to 18.3%, greatly improving the precipitation strengthening effect. Simultaneously, the dislocation density increases from 5.01 × 10^13^/m^2^ to 1.39 × 10^14^/m^2^, improving the strengthening effect, as shown in Figure 12. Therefore, the tensile strength of Cu-0.12Mg samples is over 150 MPa greater than that of Cu-0Mg samples. However, the addition of 0.24%Mg has little effect on the number of precipitates, particularly small precipitates, so that the strength does not change much, but the electrical conductivity decreases significantly. Qi [43] found that a high number of primary precipitates may suppress the nucleation and promote the growth of the precipitation phase after rolling. The addition of 0.24%Mg may lead to an increase in the content of primary precipitates, resulting in a degree of suppression of precipitate nucleation after aging, while the proportion of small precipitates is not further increased.

## 5. Conclusions

This paper investigated the effect of aging treatment on the properties of cold-rolled Cu-Ni-Si-Co alloys with different Mg contents. The evolution of the (Ni, Co)_2_Si precipitates with Mg addition and aging treatment were observed. The dislocation density of the Cu-Ni-Si-Co-Mg alloys was calculated based on a modified Williamson–Hall formula and XRD measurements. The cold rolling process described in this paper is straightforward to operate and highly feasible in actual production. It also has good industrial scalability, enabling the addition of hot rolling or multi-pass cold rolling processes and the exploration of cost-effective manufacturing methods.

The following conclusions were drawn:

(1) The precipitate was (Ni, Co)_2_Si and exhibited a strip shape. For the Cu-0.12Mg-300 °C samples, a substantial quantity of small (Ni, Co)_2_Si precipitates were formed in the early stage of aging. However, as the aging time increased, the coarsening of the precipitates became increasingly dominant. The addition of 0.12%Mg promoted the precipitation of small precipitates. The number of precipitates increased by 6.6%, while the average particle size decreased by 11.8%. However, the number of precipitates increased by just 1.2% as the Mg content increased from 0.12% to 0.24%.
(2) For the Cu-0.12Mg-300 °C samples, the dislocation density showed an increasing and then decreasing trend with the aging time. The value increased from 1.2 × 10^14^/m^2^ (at 0 min) to 3.38 × 10^14^/m^2^ (at 20 min) and then decreased to 1.39 × 10^14^/m^2^ (at 40 min). The aging temperature had a significant effect on the dislocation density, which decreased from 1.39 × 10^14^/m^2^ to 2.21 × 10^13^/m^2^ as the aging temperature increased from 300 °C to 400 °C. The addition of 0.12%Mg resulted in a corresponding increase in the dislocation density.
(3) The addition of 0.12%Mg increased the tensile strength by around 150–200 MPa, but caused a slight reduction in the electrical conductivity. However, as the Mg content increased from 0.12% to 0.24%, there was little change in tensile strength, but the electrical conductivity decreased significantly. The electrical conductivities of the Cu-0Mg-300 °C-20 min, Cu-0.12Mg-300 °C-20 min and Cu-0.24Mg-300 °C-20 min samples were 43.4%IACS, 42.2%IACS and 39.5%IACS, respectively. Meanwhile, their respective tensile strengths were 489 MPa, 685 MPa and 663 MPa. The Cu-0.12Mg-300 °C-20 min sample had the best performance in terms of overall electrical conductivity and tensile strength.

## Figures and Tables

**Table 1:** Alloying element contents in Cu alloys (wt.%).

No.	Alloy	Ni	Co	Si	Mg	Cu
Cu-0Mg	Cu-1.86Ni-1.1Co-0.6Si-0Mg	1.82	1.05	0.6	0	Bal.
Cu-0.12Mg	Cu-1.86Ni-1.1Co-0.6Si-0.12Mg	1.82	1.06	0.59	0.12	Bal.
Cu-0.24Mg	Cu-1.86Ni-1.1Co-0.6Si-0.24Mg	1.90	1.08	0.61	0.23	Bal.

## Data Availability

The original contributions presented in this study are included in the article. Further inquiries can be directed to the corresponding authors.
